# Tracking the Origin of Austrian Human Brucellosis Cases Using Whole Genome Sequencing

**DOI:** 10.3389/fmed.2021.635547

**Published:** 2021-02-24

**Authors:** Justine Schaeffer, Sandra Revilla-Fernández, Erwin Hofer, Romana Posch, Anna Stoeger, Christoph Leth, Friedrich Schmoll, Vesna Djordjevic, Brankica Lakicevic, Kazimir Matovic, Peter Hufnagl, Alexander Indra, Franz Allerberger, Werner Ruppitsch

**Affiliations:** ^1^Institute for Medical Microbiology and Hygiene, Austrian Agency for Health and Food Safety (AGES), Vienna, Austria; ^2^EUPHEM Fellowship, European Centre for Disease Prevention and Control (ECDC), Stockholm, Sweden; ^3^Institute for Veterinary Disease Control Mödling, Austrian Agency for Health and Food Safety (AGES), Mödling, Austria; ^4^Department of Microbiology and Molecular Biology, Institute of Meat Hygiene and Technology, Belgrade, Serbia; ^5^Department for Laboratory Diagnostic, Veterinary Specialized Institute, Kraljevo, Serbia

**Keywords:** brucellosis, *Brucella melitensis*, whole genome sequencing, core genome multilocus sequence typing, imported case

## Abstract

Brucellosis is a zoonotic disease caused by *Brucella* spp. and a major concern for livestock. Most human cases are caused by *B. melitensis* and clinical presentation is usually a mild febrile illness. However, treatment failure is frequent and more severe complications can occur. In Austria, every human brucellosis is investigated to determine whether it was imported from endemic areas or is the sign of an undetected autochthonous transmission. For this study, 21 *B. melitensis* strains isolated in Austria between 2005 and 2019 were collected, 17 strains from 15 different patients and four strains from cattle. Whole genome sequencing combined with core-genome MLST analysis was used to characterize these strains. A cluster of seven isolates from 2018 (three human and four cattle isolates) was identified, with fewer than two allelic differences. They corresponded to the only Austrian *B. melitensis* outbreak that happened over the past 15 years. The other 12 Austrian brucellosis cases were single cases, and geographical origins were available for 8/12. Genomic data was used to locate probable geographical origins and compared with the results of the epidemiological investigations. Austrian strains were compared with 67 published *B. melitensis* sequences available on NCBI. The result of genomic analysis matched for 7/8 cases with documented conclusion of the epidemiological investigation. Genome analysis also pointed to the geographical origin for three of the four cases with missing epidemiological data. Strains from six cases were grouped together (<40 allelic differences) with 4/6 cases imported from the Balkans. Additional *B. melitensis* isolates from Serbian animals were analyzed and grouped with this branch, suggesting frequent importation from Balkan countries to Austria. Overall, this study highlights the specificities of human brucellosis in Austria. It also underlines the value of whole genome sequencing as a tool to investigate brucellosis cases, allowing to identify and investigate outbreaks but also to support epidemiological investigation of imported cases. However, the reliability of such methods depends on the number of strains for comparison, which can be challenging in low incidence countries. Increasing the availability of published sequences with documented geographical origins would help establishing genomic-based methods for investigating brucellosis cases.

## Introduction

Brucellosis is a zoonotic disease caused by *Brucella* spp. Four species are responsible for most human brucellosis cases, and each one is usually associated with a different animal reservoir: *B. melitensis* (sheep and goat), *B. abortus* (cow), *B. suis* (pig), and *B. canis* (dog) (1). *B. melitensis* is the predominant species causing human brucellosis, and is associated with more severe disease (2). In sheep and goat, brucellosis is usually a subacute or chronic infection of the reproductive system, but can result in abortions in pregnant females (2). These abortions make brucellosis a leading cause of economic losses in the livestock sector (3). *B. melitensis* can be transmitted to humans via direct contact with infected animals or consumption of contaminated food (mainly dairy products) (4). Clinical presentation is usually an unspecific mild febrile illness that can be difficult to diagnose (5). Failure of early treatment often leads to bacterial spread and disease progression in the genitourinary, neurological, pulmonary, or cardiovascular systems (6). The most serious complication is endocarditis, reported in 2% of cases (2). Human brucellosis treatment is tedious, the recommended protocol consisting of two combined antibiotics for at least 6 weeks (7). Even with antibiotic treatment, the disease may persist as relapse, chronic localized infection or delayed convalescence (2).

Brucellosis is one of the world's most widespread zoonosis, with an estimated incidence of 500,000 cases per year (3). It is considered a re-emerging disease, highly spread by traveling and globalization (8). In Europe, brucellosis is rare in both cattle and humans (9). Most EU countries have eradicated bovine and ovine/caprine brucellosis, preventing food-borne outbreaks. However, over 380 cases of human brucellosis, mainly *B. melitensis* infections, were reported in the EU in 2017 and more than 60% required hospitalization (4). In cattle, Austria is officially brucellosis-free (OBF), but a few human cases are still reported every year (10). The incidence is lower than the EU average, with 0.05 cases/100,000 inhabitants. To maintain this OBF status, every human brucellosis is reported and investigated to identify the source of infection. In the past years most investigations found only imported cases, until a local outbreak was identified in 2018.

As *B. melitensis* infections in Austria are rare and usually imported, collecting as many details as possible is essential to better understand patterns of transmission. This includes epidemiological information on patient's history and also microbiological data on bacterial strains. Whole genome sequencing (WGS) allows to study the entire genetic information of the strain and is therefore highly discriminatory (11). In species with low genetic diversity such as *B. melitensis*, it is the easiest way to accurately type isolates which is highly valuable to identify the source of infection. Additional data, such as the identification of antimicrobial resistance (AMR) genes, can also be extracted from WGS data. Molecular surveillance of *B. melitensis* allows to investigate precisely every case, human and animal.

In this study, WGS was used to characterize Austrian *B. melitensis* isolates. The sequences of the isolates were compared with published genomes of strains from various countries. Similarities between the Austrian and published sequences were estimated using classical multi-locus sequence typing (MLST), but also stable core genome MLST (cgMLST) (12). This analysis provided information on the clustering of the Austrian isolates and comparison with each other but also with published sequences from various geographical areas. Additional data on the possible origins of the strains would allow further study of the patterns of *B. melitensis* importation to Austria.

## Materials and Methods

### Isolates

Between 2005 and 2019, 21 *B. melitensis* strains were received by the Austrian National Reference Center (NRC) for brucellosis for confirmation and species identification. Among these strains, 17 were human clinical isolates (blood or pus from an epidural abscess) and four were isolated from cattle (Table 1). Clinical, epidemiological and microbiological data used in this study fell within the legal mandate given to the NRC by the Austrian Ministry of Health and did not require additional ethical approval. Informed consent was not required because of the retrospective design of this study.

**Table 1:** Sequencing data, quality data, and accession numbers of the *B. melitensis* isolate genomes.

**ID**	**Isolation source**	**ST**	**Genome size (Mb)**	**No. of reads**	**Avg. coverage**	**No. of contigs**	**Contig N50 (bp)**	**G+C content (%)**	**Total no. of genes**	**No. of RNAs**	**cgMLST good targets (%)**	**GenBank accession no**.	**SRA accession no**.
**803877**	Blood	11	3.3	2,097,558	97	102	248,235	57.12	3,180	59	99.3	JACKQR000000000	SRR13442698
**804759**	Blood	11	3.3	654,332	26	177	170,143	57.55	3,310	60	99.3	JACKQQ000000000	SRR13495169
**805594**	Blood	11	3.3	1,032,760	28	84	108,381	55.42	3,180	57	98.8	JACKRJ000000000	SRR13495168
**511496**	Blood	8	3.4	1,458,932	61	186	251,023	56.77	3,195	59	99.6	JACKRJ000000000	SRR12272640
**511497**	Blood	8	3.3	2,159,370	91	121	251,207	56.91	3,184	59	99.6	JACKRI000000000	SRR12272639
**511498**	Placenta	11	3.4	1,787,914	76	293	158,283	56.75	3,183	59	99.3	JACKRH000000000	SRR12272630
**511500**	Milk	11	3.5	1,411,980	60	301	142,342	56.83	3,187	59	99.3	JACKRG000000000	SRR12272629
**511501**	Milk	11	3.3	1,749,842	72	109	179,327	57.04	3,191	59	99.3	JACKRF000000000	SRR12272628
**511502**	Milk	11	3.4	1,381,908	62	282	159,151	56.96	3,192	59	99.1	JACKRE000000000	SRR12272627
**511503**	Blood	8	3.3	1,835,848	78	63	276,438	57.00	3,185	59	99.3	JACKRD000000000	SRR12272626
**511504**	Blood	8	3.3	1,508,082	59	48	251,121	57.06	3,187	59	99.7	JACKRC000000000	SRR12272625
**511505**	Blood	11	3.3	1,834,026	75	44	245,778	56.93	3,183	59	99.3	JACKRB000000000	SRR12272624
**511506**	Blood	8	3.3	1,100,006	47	60	189,612	57.13	3,185	59	99.5	JACKRA000000000	SRR12272623
**511507**	Epidural abscess	8	3.3	1,602,036	68	113	159,959	57.00	3,185	59	99.3	JACKQZ000000000	SRR12272638
**511508**	Blood	8	3.4	2,565,494	95	181	159,951	57.20	3,183	59	99.6	JACKQY000000000	SRR12272637
**511509**	Blood	8	3.3	1,159,170	47	68	221,658	57.05	3,178	59	99.7	JACKQX000000000	SRR12272636
**511510**	Blood	8	3.4	1,428,252	61	258	136,861	57.12	3,181	59	99.6	JACKQW000000000	SRR12272635
**511511**	Blood	8	3.3	3,032,674	100	101	156,092	57.18	3,179	59	99.4	JACKQV000000000	SRR12272634
**511512**	Blood	8	3.3	780,376	37	296	55,938	56.71	3,216	58	98.2	JACKQU000000000	SRR12272633
**511513**	Blood	11	3.3	1,431,630	61	54	189,859	57.14	3,186	59	99.4	JACKQT000000000	SRR12272632
**511514**	Blood	8	3.3	1,018,898	42	55	251,061	56.46	3,184	59	99.5	JACKQS000000000	SRR12272631
**510332**	Blood	8	3.3	2,374,878	113	57	251,039	57.03	3,178	59	99.6	JACKQR000000000	SRR12420834
**510333**	Blood	8	3.3	2,709,082	123	79	296,915	57.33	3,177	59	99.6	JACKQQ000000000	SRR12420833

### Whole Genome Sequencing

For WGS genomic DNA isolation, library preparation, sequencing run, assembly, and contig filtering were performed as described previously (13). Briefly, high-molecular-weight DNA was isolated from a culture using MagAttract HMW DNA Kit (QIAGEN, Hilden, Germany), following the manufacturers' protocol for Gram-negative bacteria. Library preparation to obtain ready-to-sequence libraries was done with a NexteraXT kit (Illumina, Inc., San Diego, CA, USA). Paired-end sequencing (2 × 300 bp) was performed using a MiSeq system (Illumina, Inc.) and raw reads were *de novo* assembled into a draft genome using SPAdes (version 3.11.1) (14). Contigs were filtered for a minimum coverage of 5 and minimum length of 200 bp. Sequencing generated 654,332–3,032,674 reads and a mean coverage of 26 to 123-fold (Table 1). Assemblies were analyzed with the NCBI prokaryotic genome automatic annotation pipeline (Table 1).

### Sequence Analysis

SeqSphere + (Ridom GmbH, Münster, Germany) was used for isolate characterization. Two publicly available typing schemes available on the SeqSphere database, the 9 loci *B. melitensis* MLST scheme (15) and the *B. melitensis* cgMLST scheme (2,704 targets in core genome and 360 targets in accessory genome) were used (12). Minimum Spanning Tree (MST) and Neighbor Joining Tree (NJT) were made with SeqSphere.

Sixty-seven published *B. melitensis* sequences were obtained from GenBank or the Sequence Read Archive (SRA, Supplementary Table 1) (16). Accessing the National Center for Biotechnology Information (NCBI) from Seqsphere, 63 assembled complete genomes of *B. melitensis* were found. The 51 belonging to ST8 (49 strains) and ST11 (2 strains) were included. As more ST11 sequences were needed for our analysis, our search was extended for scaffold and contig assemblies. Thirteen ST11 published sequences with documented origin could be added. Finally, some ST11 Italian strains were added because of the geographic proximity of Austria and Italy. From the Janowicz et al. study (12), one sheep isolate from a 2015–2016 outbreak in Molise and two human isolates from northern Italy (Piedmont, 2015 and 2017) were added. For these three isolates, read files were downloaded from SRA and assembled using SPAdes.

Single nucleotide polymorphism (SNP) analysis of sequences was performed with CSI Phylogeny 1.4, available from the Center for Genomic Epidemiology (17), using the strain 16M as a reference genome (NC_003317.1 and NC_003318.1). Dendrograms were created from distance matrix (of SNP analysis or cgMLST) with R (version 3.6.1) using packages msa and dendextend.

#### Data Availability

Raw sequence reads and assemblies were deposited in the SRA and in the GenBank database as project PRJNA647424. Biosamples were assigned accession nos. SAMN15586354 to SAMN15586371, SAMN15774184, SAMN15774185, SAMN17320939, SAMN17393919, and SAMN17393920. SRA accessions no. of raw sequences and GenBank accession no. assigned to assemblies are indicated in Table 1.

## Results

### Samples and WGS

Twenty-one strains were analyzed in this study including four strains isolated from cattle (three from raw milk samples and one from a placenta tissue sample) and 17 strains isolated from 15 different patients (Table 2). These brucellosis cases were reported between 2005 and 2019. Two patients had two isolates each (511509/511510 and 511496/511497). For 13 cases clinical data were available in the register of mandatorily reportable diseases. These patients were mostly adults (12/13) and male (9/13). This is in accordance with usual brucellosis patterns of contamination with adult men being predominantly affected (4). The clinical presentation was mostly fever and fatigue. The delay between date of onset and date of microbiological investigation (or reception date in Table 2) was between eight and 34 days (mean: 24 days; median: 18 days). However, given the low specificity of symptoms, date of onset can be subject to recall bias. In 11/15 cases epidemiological investigation documented imported etiology with the suspected country of infection.

**Table 2:** Patient and epidemiological data corresponding to the *B. melitensis* isolates.

**ID**	**Date of reception at NRC**	**Austrian state of isolation**	**Age**	**Sex**	**Nationality**	**Symptoms**	**Date of disease onset**	**Suspected country of infection**
511511	01.01.2005	Vienna	NA	NA	NA	NA	NA	NA
511513	01.08.2008	Vienna	NA	NA	NA	NA	NA	NA
511508	18.10.2011	Styria	46	M	Austrian	headache, weakness	19.09.2011	Bosnia-Herzegovina
511509	24.10.2011	Vienna	77	M	Austrian	NA	10.10.2011	Austria (Turkish food)
511510	30.11.2011	Vienna				NA		
511507	20.02.2012	Vienna	47	M	NA	NA	NA	NA
511512	04.07.2012	Lower Austria	35	F	Austrian	fever, chills	01.06.2012	Serbia
511505	12.07.2013	Upper Austria	34	M	Austrian	fever, weakness	NA	NA
511506	05.12.2013	Vienna	54	F	Austrian	fever, weakness	01.11.2013	Turkey
511503	15.01.2016	Tyrol	33	M	Afghan	arthralgia, fever	31.12.2015	Afghanistan
511504	11.07.2016	Vienna	65	M	Croatian	arthralgia, fever	23.06.2016	Croatia
511496	08.05.2017	Tyrol	46	M	Bosnian	fever	27.04.2017	Bosnia-Herzegovina
511497	02.09.2019	Tyrol	49			arthralgia	25.08.2019	
803877	21.06.2018	Upper Austria	46	M	Austrian	fever, weakness	06.06.2018	Austria
804759	30.07.2018	Upper Austria	6	F	Austrian	fever	20.07.2018	Austria
805594	07.09.2018	Upper Austria	38	M	Austrian	fever, chills	23.08.2018	Austria
511514	28.01.2019	Lower Austria	30	F	Austrian	anorexia, arthralgia, fever, chills, weakness	unknown	Turkey

Draft genomes obtained by *de novo* assembly consisted of 44–186 contigs. The NCBI Prokaryotic Genome Automatic Annotation Pipeline identified 3,177–3,310 genes, 2,964–3,008 coding sequences, 57–60 RNAs, 133–161 pseudogenes, 4 rRNA genes and 51−52 tRNA genes (Table 1). Sequences were analyzed for AMR genes using the CARD database (18). Assemblies from all the isolates contained *B. suis* mprF, which confers AMR to defensins. No other strict hit for resistance genes was found in any of the isolates.

### Comparison of Austrian Strains

To compare *B. melitensis* isolates with each other, MLST typing was first used, extracting sequence types (ST) from WGS data (Table 1). The 21 strains belonged to 2 STs: ST8 (12 strains) and ST11 (9 strains). Previously isolated ST11 *B. melitensis* strains were of European origin, whereas ST8 strains had been isolated all over the world. Obtaining only 2 different ST for 21 strains isolated between 2005 and 2019 underlines the low discriminatory power of MLST for *B. melitensis* typing and the need for better alternatives. Therefore, a genome wide approach such as cgMLST seemed to be more appropriate.

**Figure 1:**
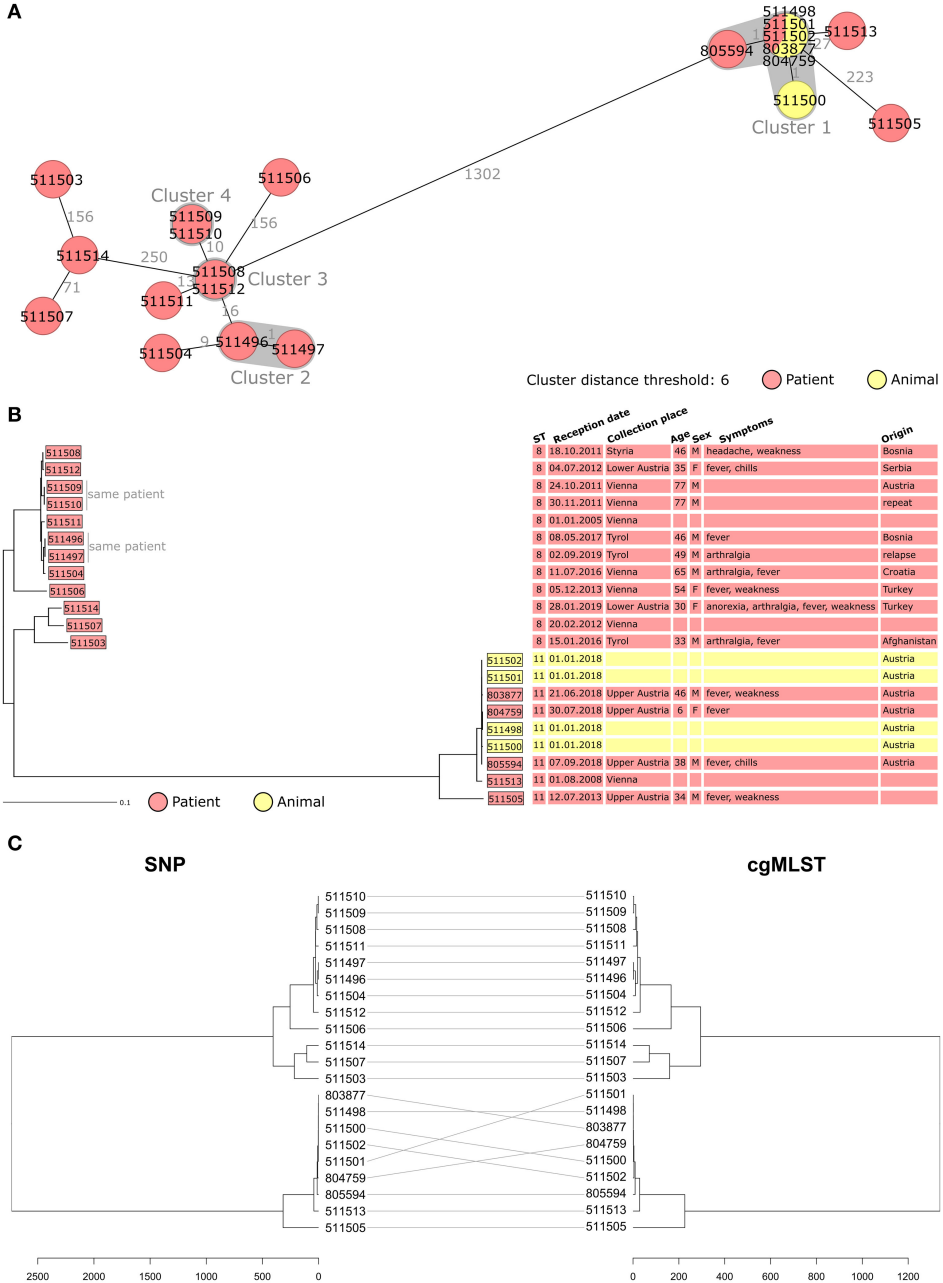
**(A,B)** Minimum spanning tree [MST, **(A)**] and Neighbor Joining Tree [NJT, **(B)**] of *B. melitensis* isolates obtained in Austria between 2005 and 2019 (*N* = 21). MST and NJT were computed using the number of allelic differences in stable *B. melitensis* cgMLST. Colors indicate the type of sample from which strain was isolated. **(A)** Isolate IDs are indicated on the nodes. Distance between the isolates (in number of allelic differences) are indicated on the connecting lines. Clusters with fewer than 6 allelic differences are shaded in dark gray and named (cluster 1, cluster 2, etc.). **(B)** Scale bar indicates the percentage of allelic differences between the isolates. Columns on the right provide additional information on isolates: ST, sample information and patient data. **(C)** Dendrogram computed from the distance matrix of single nucleotide polymorphism analysis (left) and cgMLST analysis (right). Gray lines connect isolates in both trees. Scale bar indicates the number of differences between the isolates, in nucleotides (left) or allelic differences (right).

The Janowicz cgMLST scheme was used to compare the Austrian *B. melitensis* isolates (Figures 1A,B) (12). Isolates segregated in two groups separated by a minimum of 1,302 allelic differences which corresponded to ST8 and ST11 strains (Figure 1A). Among ST11 isolates, the four cattle strains and three clinical isolates clustered together. They corresponded to an Austrian outbreak detected at a dairy farm in May 2018 (19). Following an epidemic of miscarriages, *B. melitensis* was isolated from aborted bovine material (isolate 511498) and milk (isolates 511500, 511501, and 511502). Two veterinarians and the farmer's child were hospitalized and diagnosed with brucellosis (isolates 803877, 804759, and 805594). The genetic similarity between the cattle and human isolates (<2 allelic differences) confirmed that they all belonged to the same outbreak. It proved the first zoonotic transmission of *B. melitensis* in Austria in more than 15 years. During the outbreak investigation, three additional cases were identified using serology. Patient isolate 511513 from 2008 had only 27 allelic differences to the outbreak cluster of 2018, indicating that the strain responsible for the 2018 outbreak might have been circulating undetected for a longer time (Figure 1B).

Among the 12 isolates corresponding to ST8, eight (from six human cases) grouped together with fewer than 40 allelic differences. Among them, two clusters were identified (cluster 2 and cluster 3, Figure 1A) and corresponded to repeated sampling from the same patient. For cluster 3, the second isolate was collected 1 month after the first one to test for treatment efficacy and was identical in cgMLST. For cluster 2, the two isolates were obtained 2 years apart (2017 and 2019). After being cured of brucellosis in 2017 this patient developed a second episode of brucellosis in 2019. WGS analysis of the 2019 isolate showed only one allelic difference to the 2017 strain.

To assess whether the discriminatory power of cgMLST was sufficient to efficiently compare *B. melitensi*s isolates, SNP analysis was performed. The alignments obtained with SNP and cgMLST (Figure 1C) were compared. Both analyses showed identical clustering of the Austrian isolates. This result confirmed that cgMLST was powerful enough for *B. melitensis* strain typing, and it was therefore used for the rest of the study.

### Comparison With Published Sequences

**Figure 2:**
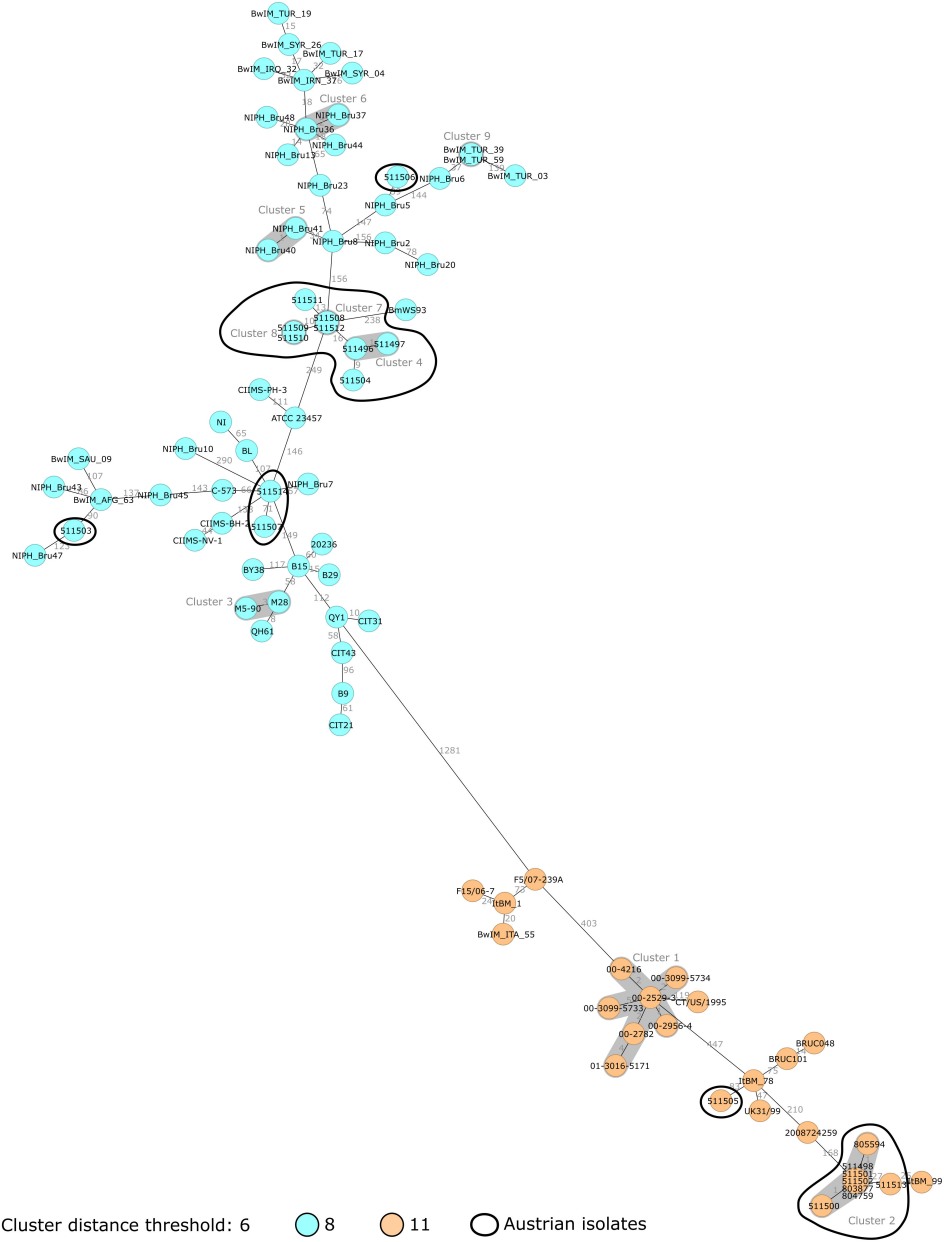
Minimum spanning tree (MST) of *B. melitensis* isolates obtained in Austria between 2005 and 2019 (*N* = 21, circled in black) and publicly available sequences (*N* = 67). MST was computed using the number of allelic differences in stable *B. melitensis* cgMLST. Isolate IDs are indicated on the nodes. Distance between the isolates (in number of allelic differences) are indicated on the connecting lines. Nodes are colored according to isolate ST. Clusters with fewer than 6 allelic differences are shaded in dark gray and named (cluster 1, cluster 2, etc.).

To investigate the divergence between the Austrian *B. melitensis* strains and the epidemiological relevance, it was decided to compare them with published sequences. Forty-nine ST8 strains and 18 ST11 strains were included in the analysis (Supplementary Table 1). Published sequences corresponding to ST8 strains were more abundant, as ST8 is a common ST. ST8 and ST11 isolates grouped separately, with a minimum of 1,281 allelic differences (Figure 2). Two groups of Austrian isolates (one ST8 group and one ST11 group) were identified.

**Figure 3:**
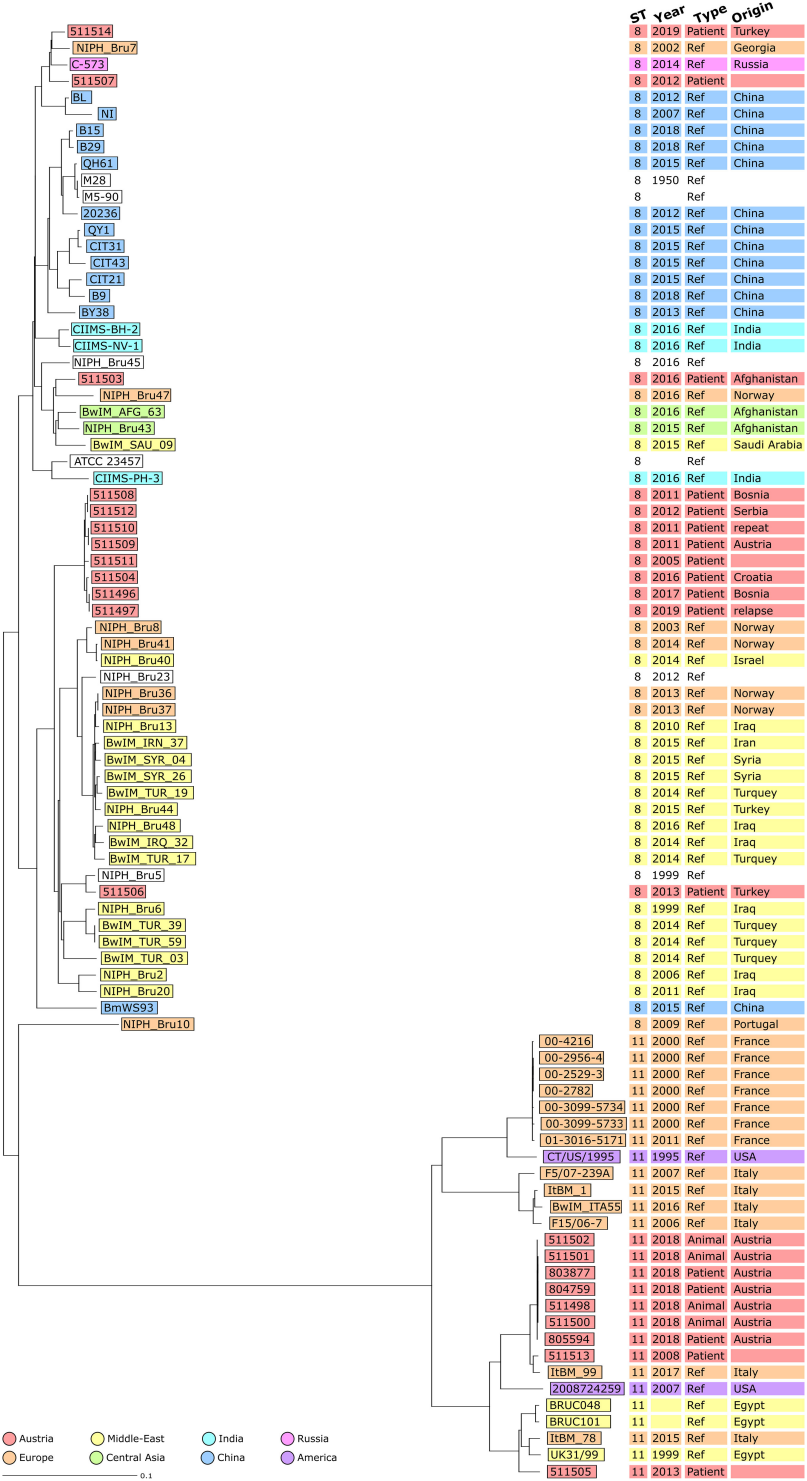
Neighbor Joining Tree (NJT) of *B. melitensis* isolates obtained in Austria between 2005 and 2019 (*N* = 21) and publicly available sequences (*N* = 67). NJT was computed using the number of allelic differences in stable *B. melitensis* cgMLST. Scale bar indicates the percentage of allelic differences between the isolates. Colors indicate isolate geographical region of origin. Columns on the right provide additional information on isolates: ST, collection date of the sample, type of sample used for isolation and country of infection.

In Figure 3, Austrian isolates and published sequences were compared using a NJT and colored depending on the reported region of origin. Isolate 511506 grouped with Turkish strains, and epidemiological investigation concluded that it was imported from Turkey (with documented exposure to raw cheese in Turkey). This isolate showed concordance between epidemiological investigations and genomic clustering. Isolate 511503 corresponded to an Afghan patient with reported travel from Afghanistan but no documented exposure (neither contact with animals nor consumption of raw food). This isolate grouped with other strains from central Asia supporting the hypothesis of having been imported from Afghanistan. Isolates 511507 and 511514 grouped with strains from Russia and Georgia, which corresponded to an eastern Mediterranean region clade. As only two NCBI strains belonged to this branch, no global conclusion could be drawn. However, for isolate 511514, the epidemiological investigation highlighted exposure to raw milk in both Turkey and Austria. The genetic data indicates Turkey as the more likely source of infection than Austria.

The ST11 strains formed a different branch compared to other STs (Figure 3). The seven isolates from the 2018 Austrian outbreak clustered together. Strain 511505 belonged to a branch including three strains from Egypt and an Italian strain of unknown epidemiological status. This suggested that both the Austrian case corresponding to isolate 511505 and also the Italian case corresponding to isolate ItBM_78, were imported from Egypt. Strain 511513 was closer to an Italian clinical strain from Turin (25 allelic differences) as well as the 2018 Austrian outbreak strains (27 allelic differences).

The group of eight ST8 Austrian strains that was identified previously did not cluster with other strains from the NCBI (Figure 3). Among these patient isolates, four out of six cases were suspected to have been infected in Balkan countries. The two patients corresponding to isolates 511508 and 511512 had documented exposure to raw food (unpasteurized milk in Bosnia and raw cheese in Serbia) supporting the conclusion of the epidemiological investigation that these cases were imported. However, for isolates 511496/511497 and 511504, patients had traveled to Bosnia and Croatia but had no documented exposure to animals or raw food. For isolates 511509/511510, the patient did not report travel to endemic countries, but consumed food imported from Turkey in Austria. Altogether, on the assumption of an importation route from the Balkans to Austria, some of these cases would require further investigation.

**Figure 4:**
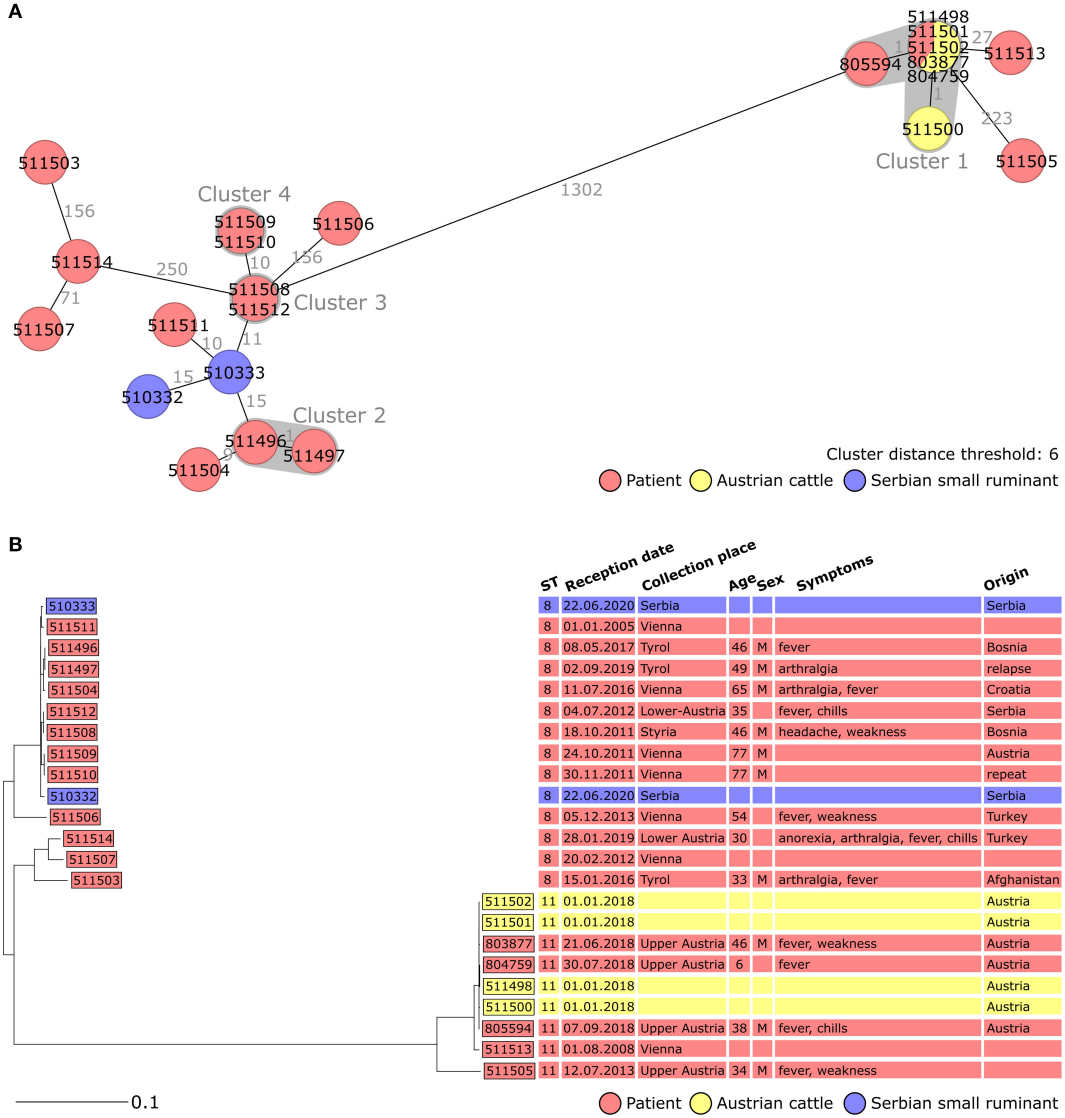
Minimum spanning tree [MST, **(A)**] and Neighbor Joining Tree [NJT, **(B)**] of *B. melitensis* isolates obtained in Austria between 2005 and 2019 (*N* = 21, red and yellow) and isolates obtained from Serbian animals (*N* = 2, blue). MST and NJT were computed using the number of allelic differences in stable *B. melitensis* cgMLST. **(A)** Isolate IDs are indicated on the nodes. Distance between the isolates (in number of allelic differences) are indicated on the connecting lines. Clusters with fewer than 6 allelic differences are shaded in dark gray and named (cluster 1, cluster 2, etc.). **(B)** Scale bar indicates the percentage of allelic differences between the isolates. Columns on the right provide additional information on isolates: ST, sample information and patient data.

To test the hypothesis that these six cases were imported from the Balkans, additional *B. melitensis* strains from these countries were requested. Serbia provided two strains isolated from sheep and goats which were included in the analysis (Figure 4). They also grouped with the eight Austrian isolates. This finding supported the hypothesis that these eight Austrian strains, including those from patients with no reported exposure to the Balkans, might have been imported from this region. If this conclusion was in accordance with the epidemiological investigation for isolates 511496/511497 and 511504, it is not the case for isolate 511509/511510. In this case, the assumed contamination via imported Turkish food was not supported by the WGS analysis. Overall, these results also highlight that a large proportion of Austrian brucellosis cases were linked to the Balkans.

## Discussion

Although the incidence of human brucellosis in Austria is very low, with fewer than 10 cases per year, the individual burden of the disease makes it a relevant public health problem (10). In 2014, an exploratory nationwide cross-sectional seroprevalence survey in 526 healthy adults was conducted and all individuals tested negative for antibodies against *B. melitensis/B. abortus* (20). A solid surveillance system tracks brucellosis cases in both humans and animals and every case has to be reported and confirmed by the NRC for brucellosis. Human cases are investigated to find the source of infection and prevent further cases. Identifying local outbreaks and segregating them from imported cases is of high importance to maintain Austria's OBF status.

In this study, WGS was used to characterize 21 *B. melitensis* strains isolated in Austria since 2005. WGS data were searched for AMR genes and only one gene conferring AMR to defensins (*B. suis* mprF) was found. This gene usually belongs to the resistome of *Brucella* spp and was identified in over 99% of *B. melitensis* WGS from NCBI. Testing AMR in *B. melitensis* is rarely included in surveillance, because it requires bacterial culture in a BLS3 laboratory. Using WGS data to screen for AMR genes could be a convenient alternative, although it is not as informative as proper phenotypical AMR testing. Among the 21 Austrian isolates, 17 strains were isolated from 15 different patients. Two patients had repeated sampling, 1 month apart for the first case and 2 years apart for the second. In both cases, the two strains were similar, reducing the possibility of two independent infections. For the first patient, the fact that bacteria could still be isolated after 1 month of treatment illustrated the potential for *B. melitensis* persistence. For the second case, the second episode of brucellosis was concluded to be a relapse, with the bacteria persisting in the organism for 2 years.

Isolate typing was performed using cgMLST. One major cluster was identified including three human and four cattle isolates. They corresponded to an autochthonous outbreak identified in 2018. WGS had enabled detection of this outbreak, showing that three patients diagnosed over a period of 3 months were infected by the same *B. melitensis* strain (1 allelic difference). It underlines how useful genetic comparison of strains (using standard MLVA-typing or more recently WGS) can be to support outbreak investigations in real time (19, 21). A retrospective study in Portugal even identified likely “missed outbreaks” using WGS, as isolates with no documented links clustered (22). When environmental samples are available, comparing isolates using genetic techniques can also confirm the source of infection. It was the case in the 2018 Austrian outbreak, where *B. melitensis* isolates from cattle placenta and milk clustered with the three clinical isolates. For such investigations, it is worth implementing molecular techniques with the highest possible discriminatory power because they provide a higher probability that two isolates, with similar genetic profiles, are indeed related.

With the exception of the three cases from the 2018 outbreak mentioned above, the other 12 human cases included in this study were single cases. For 8/12, the conclusion of the epidemiological investigation regarding the source of infection was documented. However, for the other 4 cases, epidemiological data were incomplete and no data on whether or not the cases were imported was available. Therefore, it was decided to use the genomic data to identify the most likely geographical origins of the cases. This could have been achieved using environmental or animal samples, as done in a study from Germany where they collected *B. melitensis* strains from Turkish sheep (23). They were able to link these animal isolates to clinical isolates from travelers returning from Turkey or Turkish immigrants, implying that these cases were indeed imported from Turkey. However, neither food nor animal samples were collected for any of the 12 Austrian patients. The next option was to compare Austrian *B. melitensis* strains with each other, analyzing how they clustered together. Previous studies used this method, but they had access to a large number of isolates (11, 12). Among the 21 Austrian isolates, one branch with a high proportion of cases imported from the Balkans was identified, but the low number of cases prevented any strong conclusions.

To counterbalance the limitations due to the low sample size, publically available sequences were included in the analysis. This method has proven useful to indicate the geographical origin of brucellosis cases in previous studies, using either MLVA-typing or WGS (24–26). As shown in the study from Sacchini et al., (27) which compared MLST, MLVA and cgMLST to analyze *B. melitensis* isolates, higher throughput techniques give results with higher discriminatory power (27). In our WGS-based study, the clustering of seven Austrian isolates with published sequences corroborated the country of infection found by the epidemiological investigation. For the four remaining patients it allowed us to suggest a plausible origin; the confidence of this conclusion depends on the number of publically available sequences. This is one of the biggest limitations of this method: its accuracy depends strongly on the number of published sequences with reliable metadata (11). Increasing the pool of available *B. melitensis* sequences and better data concerning geographical origins would allow WGS-typing to better support epidemiological investigations.

One of the most interesting findings of this study was the identification of a group of isolates originating in the Balkans. The group included cases imported from Balkan countries and also two animal isolates provided by Serbia. This finding was quite unusual among EU countries. Previous studies from Belgium, Germany, Norway and Sweden have highlighted a high proportion of imported cases originating from the eastern Mediterranean region (11, 25–27). Such differences might rely on the geopolitical situation of Austria which has tighter bonds with Eastern Europe and Balkan countries compared to other EU member states. Enhanced international collaboration between Austrian health authorities and their Balkan counterparts would allow a better understanding of these patterns of importation. It could also suggest targeted control measures to reduce the incidence of brucellosis, both in Austria and in the Balkans.

In conclusion, this study highlights several specificities of brucellosis in Austria. It relied on WGS, which is a well-adapted tool for *B. melitensis* typing thanks to its high discriminatory power. WGS has proven valuable to identify and investigate outbreaks. In addition, comparing large batches of sequences could help identify possible geographical origins of the strains. For countries with low brucellosis incidence, such techniques require an external data source. More publicly available sequences with documented origin need to be available in order to increase the reliability of this method. Overall, this study underlines the usefulness of implementing WGS for routine *B. melitensis* surveillance, in both human and animal health. It also advocates publishing *B. melitensis* sequences with relevant metadata thus increasing the pool of available genomic data.

## Data Availability Statement

The datasets presented in this study can be found in online repositories. The names of the repository/repositories and accession number(s) can be found in the article/Supplementary Material.

## Ethics Statement

Ethical review and approval was not required for the study on human participants in accordance with the local legislation and institutional requirements. Written informed consent for participation was not required for this study in accordance with the national legislation and the institutional requirements.

## Author Contributions

JS and WR planned and conducted the study. SR-F, EH, RP, CL, FS, PH, and AI provided the Austrian strains and the corresponding patient data. AS sequenced the isolates. VD, BL, and KM provided the Serbian isolates. FS and FA provided funding. JS wrote the manuscript and all co-authors provided feedback on the draft. All authors contributed to the article and approved the submitted version.

## Conflict of Interest

The authors declare that the research was conducted in the absence of any commercial or financial relationships that could be construed as ass potential conflict of interest.
