# Supplementary material for: Tracking the Origin of Austrian Human Brucellosis Cases Using Whole Genome Sequencing
Source: Front Med (Lausanne). 2021 Feb 24;8:635547. doi: 10.3389/fmed.2021.635547 (PMC7943447; doi:10.3389/fmed.2021.635547)
Supplement: Supplementary file 1 [file Table_1.DOCX]

| Accesion no. NCBI | ID | N50 | % good cgMLST targets | year of isolation | country of isolation | date of publication | published by |
| --- | --- | --- | --- | --- | --- | --- | --- |
| NC_012441.1 NC_012442.1 | ATCC 23457 | 2125701 | 99.8 | NA | NA | 25.03.2009 | Virginia Bioinformatics Institute |
| NC_017244.1 NC_017245.1 | M28 | 2126133 | 99.4 | 1950 | NA | 18.03.2011 | National Key Laboratory of Veterinary Biotechnology , Harbin Veterinary Research Institute, Chinese Academy of Agricultural Sciences |
| NC_017246.1 NC_017247.1 | M5-90 | 2126451 | 98.7 | NA | NA | 22.03.2011 | National Key Laboratory of Veterinary Biotechnology , Harbin Veterinary Research Institute, Chinese Academy of Agricultural Sciences |
| NC_017248.1 NC_017283.1 | NI | 2117717 | 99.1 | 2007 | China | 06.10.2011 | China Agricultrual University |
| NZ_CP008750.1 NZ_CP008751.1 | 20236 | 2126134 | 99.3 | 2012 | China | 04.11.2015 | Shanghai JiaoTong University School of Medicine |
| NZ_CP019679.1 NZ_CP019680.1 | C-573 | 2125194 | 97.8 | 2014 | Russia | 10.02.2017 | Stavropol Plague Control Reseach Institute |
| NZ_CP018478.1 NZ_CP018479.1 | BwIM_AFG_63 | 2125986 | 99.4 | 2016 | Afganistan | 13.06.2017 | Bundeswehr, Institute of Microbiology |
| NZ_CP018486.1 NZ_CP018487.1 | BwIM_IRN_37 | 2126353 | 99.6 | 2015 | Iran | 13.06.2017 | Bundeswehr, Institute of Microbiology |
| NZ_CP018490.1 NZ_CP018491.1 | BwIM_IRQ_32 | 2126381 | 99.5 | 2014 | Iraq | 13.06.2017 | Bundeswehr, Institute of Microbiology |
| NZ_CP018504.1 NZ_CP018505.1 | BwIM_SAU_09 | 2125987 | 99.4 | 2015 | Saudi Arabia | 13.06.2017 | Bundeswehr, Institute of Microbiology |
| NZ_CP018512.1 NZ_CP018513.1 | BwIM_SYR_04 | 2126425 | 99.6 | 2015 | Syria | 13.06.2017 | Bundeswehr, Institute of Microbiology |
| NZ_CP018526.1 NZ_CP018527.1 | BwIM_SYR_26 | 2126369 | 99.7 | 2015 | Syria | 13.06.2017 | Bundeswehr, Institute of Microbiology |
| NZ_CP018540.1 NZ_CP018541.1 | BwIM_TUR_03 | 2126379 | 99.7 | 2014 | Turquey | 13.06.2017 | Bundeswehr, Institute of Microbiology |
| NZ_CP018544.1 NZ_CP018545.1 | BwIM_TUR_17 | 2126410 | 99.7 | 2014 | Turquey | 13.06.2017 | Bundeswehr, Institute of Microbiology |
| NZ_CP018546.1 NZ_CP018547.1 | BwIM_TUR_19 | 2126378 | 99.7 | 2014 | Turquey | 13.06.2017 | Bundeswehr, Institute of Microbiology |
| NZ_CP018554.1 NZ_CP018555.1 | BwIM_TUR_39 | 2126358 | 99.7 | 2014 | Turquey | 13.06.2017 | Bundeswehr, Institute of Microbiology |
| NZ_CP018560.1 NZ_CP018561.1 | BwIM_TUR_59 | 2126342 | 99.7 | 2014 | Turquey | 13.06.2017 | Bundeswehr, Institute of Microbiology |
| NZ_CP022204.1 NZ_CP022205.1 | QY1 | 2125648 | 96.1 | 2015 | China | 03.07.2017 | Lanzhou Veterinary Research Institute |
| NZ_CP022875.1 NZ_CP022876.1 | BL | 2127882 | 99.5 | 2012 | China | 22.08.2017 | China Agricultural University |
| NZ_CP022827.1 NZ_CP022828.1 | BY38 | 2126158 | 99.4 | 2013 | China | 22.08.2017 | China Agricultural University |
| NZ_CP024653.1 NZ_CP024654.1 | QH61 | 2126173 | 99.4 | 2015 | China | 13.11.2017 | Lanzhou Veterinary Research Institute |
| NZ_LT962928.1 NZ_LT962929.1 | NIPH_Bru7 | 2116992 | 99.6 | 2002 | Georgia | 01.12.2017 | NORWEGIAN INSTITUTE OF PUBLIC HEALTH |
| NZ_LT963350.1 NZ_LT963351.1 | NIPH_Bru47 | 2117000 | 99.6 | 2016 | Norway | 01.12.2017 | NORWEGIAN INSTITUTE OF PUBLIC HEALTH |
| NZ_LT962945.1 NZ_LT962946.1 | NIPH_Bru20 | 2116992 | 99.5 | 2011 | Iraq | 01.12.2017 | NORWEGIAN INSTITUTE OF PUBLIC HEALTH |
| NZ_LT962940.1 NZ_LT962941.1 | NIPH_Bru40 | 2117105 | 99.3 | 2014 | Israel | 01.12.2017 | NORWEGIAN INSTITUTE OF PUBLIC HEALTH |
| NZ_LT962910.1 NZ_LT962911.1 | NIPH_Bru37 | 2117029 | 99.6 | 2013 | Norway | 01.12.2017 | NORWEGIAN INSTITUTE OF PUBLIC HEALTH |
| NZ_LT962943.1 NZ_LT962944.1 | NIPH_Bru41 | 2117090 | 99.4 | 2014 | Norway | 01.12.2017 | NORWEGIAN INSTITUTE OF PUBLIC HEALTH |
| NZ_LT962914.1 NZ_LT962915.1 | NIPH_Bru13 | 2117044 | 99.6 | 2010 | Iraq | 01.12.2017 | NORWEGIAN INSTITUTE OF PUBLIC HEALTH |
| NZ_LT962926.1 NZ_LT962927.1 | NIPH_Bru48 | 2117062 | 99.5 | 2016 | Iraq | 01.12.2017 | NORWEGIAN INSTITUTE OF PUBLIC HEALTH |
| NZ_LT962916.1 NZ_LT962917.1 | NIPH_Bru23 | 2117031 | 99.6 | 2012 | U | 01.12.2017 | NORWEGIAN INSTITUTE OF PUBLIC HEALTH |
| NZ_LT962924.1 NZ_LT962925.1 | NIPH_Bru2 | 2117017 | 99.6 | 2006 | Iraq | 01.12.2017 | NORWEGIAN INSTITUTE OF PUBLIC HEALTH |
| NZ_LT962912.1 NZ_LT962913.1 | NIPH_Bru44 | 2117059 | 99.6 | 2015 | Turkey | 01.12.2017 | NORWEGIAN INSTITUTE OF PUBLIC HEALTH |
| NZ_LT962920.1 NZ_LT962921.1 | NIPH_Bru45 | 2116960 | 99.5 | 2016 | U | 01.12.2017 | NORWEGIAN INSTITUTE OF PUBLIC HEALTH |
| NZ_LT962930.1 NZ_LT962931.1 | NIPH_Bru43 | 2116999 | 99.4 | 2015 | Afghanistan | 01.12.2017 | NORWEGIAN INSTITUTE OF PUBLIC HEALTH |
| NZ_LT962932.1 NZ_LT962933.1 | NIPH_Bru6 | 2117009 | 99.6 | 1999 | Iraq | 01.12.2017 | NORWEGIAN INSTITUTE OF PUBLIC HEALTH |
| NZ_LT962934.1 NZ_LT962935.1 | NIPH_Bru36 | 2117054 | 99.6 | 2013 | Norway | 01.12.2017 | NORWEGIAN INSTITUTE OF PUBLIC HEALTH |
| NZ_LT962936.1 NZ_LT962937.1 | NIPH_Bru5 | 2116986 | 99.5 | 1999 | U | 01.12.2017 | NORWEGIAN INSTITUTE OF PUBLIC HEALTH |
| NZ_LT962953.1 NZ_LT962954.1 | NIPH_Bru8 | 2117003 | 99.4 | 2003 | Norway | 01.12.2017 | NORWEGIAN INSTITUTE OF PUBLIC HEALTH |
| NZ_LT962951.1 NZ_LT962952.1 | NIPH_Bru10 | 2117043 | 99.6 | 2009 | Portugal | 01.12.2017 | NORWEGIAN INSTITUTE OF PUBLIC HEALTH |
| NZ_CP025680.1 NZ_CP025681.1 | CIIMS-BH-2 | 2125700 | 99.8 | 2016 | India | 22.01.2018 | Dr. G.M. Taori, Central India Institute of Medical Sciences |
| NZ_CP026005.1 NZ_CP026006.1 | CIIMS-PH-3 | 2122768 | 99.8 | 2016 | India | 22.01.2018 | Dr. G.M. Taori, Central India Institute of Medical Sciences |
| NZ_CP029756.1 NZ_CP029757.1 | CIIMS-NV-1 | 2126131 | 99.4 | 2016 | India | 11.06.2018 | Dr. G.M. Taori, Central India Institute of Medical Sciences |
| NZ_CP025819.1 NZ_CP025820.1 | CIT21 | 2125703 | 99.3 | 2015 | China | 10.09.2018 | Brucellosis Institute of Inner Mongolia University for the Nationalities, Tongliao 028000, China |
| NZ_CP025821.1 NZ_CP025822.1 | CIT31 | 2125815 | 99.4 | 2015 | China | 10.09.2018 | Brucellosis Institute of Inner Mongolia University for the Nationalities, Tongliao 028000, China |
| NZ_CP026337 | CIT43 | NA | 99.4 | 2015 | China | 10.09.2018 | Brucellosis Institute of Inner Mongolia University for the Nationalities, Tongliao 028000, China |
| NZ_CP034103.1 NZ_CP034104.1 | BmWS93 | 2126063 | 99.5 | 2015 | China | 03.12.2018 | National Institute of Infectious Diseases Control and Prevention |
| NZ_CP035795.1 NZ_CP035796.1 | B15 | 2126219 | 99.6 | 2018 | China | 17.02.2019 | The First Affiliated Hospital, School of Medicine, Zhejiang University |
| NZ_CP035793.1 NZ_CP035794.1 | B29 | 2126260 | 99.6 | 2018 | China | 17.02.2019 | The First Affiliated Hospital, School of Medicine, Zhejiang University |
| NZ_CP035797.1 NZ_CP035798.1 | B9 | 2126176 | 99.4 | 2018 | China | 17.02.2019 | The First Affiliated Hospital, School of Medicine, Zhejiang University |
| AQKL00000000 | UK31/99 | 619145 | 99.2 | 1999 | Egypt | 27.03.2012 | Broad Institute, UK |
| DMIL00000000 | F15/06-7 | 189637 | 98.4 | 2006 | Italy | 02.11.2012 | Broad Institute, UK |
| OWOX00000000 | 00-3099-5733 | 248238 | 99.4 | 2000 | France | 02.01.2019 | ANSES, France |
| OWOP00000000 | 01-3016-5171 | 200461 | 99.4 | 2011 | France | 02.01.2019 | ANSES, France |
| OWOS00000000 | 00-2529-3 | 189847 | 99.4 | 2000 | France | 02.01.2019 | ANSES, France |
| AQNK00000000 | F5/07-239A | 1022034 | 99.2 | 2007 | Italy | 13.03.2012 | Broad Institute, UK |
| LUFK00000000 | BRUC101 | 221367 | 99.3 | NA | Egypt | 11.11.2015 | MRIGlobal, USA |
| LBFG00000000 | CT/US/1995 | 775542 | 99.4 | 1995 | USA | 16.03.2015 | MRIGlobal, USA |
| LUFL00000000 | BRUC048 | 251324 | 99.3 | NA | Egypt | 11.11.2015 | MRIGlobal, USA |
| OWOR00000000 | 00-2956-4 | 276497 | 99.5 | 2000 | France | 02.01.2019 | ANSES, France |
| OWOQ00000000 | 00-4216 | 251338 | 99.4 | 2000 | France | 02.01.2019 | ANSES, France |
| OWOT00000000 | 00-2782 | 248247 | 99.4 | 2000 | France | 02.01.2019 | ANSES, France |
| OWOW00000000 | 00-3099-5734 | 238750 | 99.4 | 2000 | France | 02.01.2019 | ANSES, France |
| SRR6958042 | ItBM_78 | 97724 | 99.1 | 2015 | Italy | 07.04.2018 | Istituto Zooprofilattico Sperimentale, Italy |
| SRR6957949 | ItBM_99 | 40314 | 98.8 | 2017 | Italy | 07.04.2018 | Istituto Zooprofilattico Sperimentale, Italy |
| SRR6958031 | ItBM_1 | 120594 | 99.2 | 2015 | Italy | 07.04.2018 | Istituto Zooprofilattico Sperimentale, Italy |

**Supplementary table 1:** data of reference sequences downloaded from the NCBI database
